# Trend of Narratives in the Age of Misinformation

**DOI:** 10.1371/journal.pone.0134641

**Published:** 2015-08-14

**Authors:** Alessandro Bessi, Fabiana Zollo, Michela Del Vicario, Antonio Scala, Guido Caldarelli, Walter Quattrociocchi

**Affiliations:** 1 IUSS Institute for Advanced Study, Pavia, Italy; 2 Laboratory of Computational Social Science, IMT Institute for Advanced Studies, Lucca, Italy; 3 ISC-CNR UoS Sapienza, Roma, Italy; 4 London Institute of Mathematical Science, London, United Kingdom; University Toulouse 1 Capitole, FRANCE

## Abstract

Social media enabled a direct path from producer to consumer of contents changing the way users get informed, debate, and shape their worldviews. Such a *disintermediation* might weaken consensus on social relevant issues in favor of rumors, mistrust, or conspiracy thinking—e.g., chem-trails inducing global warming, the link between vaccines and autism, or the New World Order conspiracy. Previous studies pointed out that consumers of conspiracy-like content are likely to aggregate in homophile clusters—i.e., echo-chambers. Along this path we study, by means of a thorough quantitative analysis, how different topics are consumed inside the conspiracy echo-chamber in the Italian Facebook. Through a semi-automatic topic extraction strategy, we show that the most consumed contents semantically refer to four specific categories: *environment*, *diet*, *health*, and *geopolitics*. We find similar consumption patterns by comparing users activity (likes and comments) on posts belonging to these different semantic categories. Finally, we model users mobility across the distinct topics finding that the more a user is active, the more he is likely to span on all categories. Once inside a conspiracy narrative users tend to embrace the overall corpus.

## Introduction

According to [1], causation is bound to the way communities attempt to make sense to events or facts. Such a phenomenon is particularly evident on the web where users, immersed in homophile and polarized clusters [2–4], process information through a shared system of meaning [5, 6]. Indeed, social media enabled a direct path from producers to consumers of contents—i.e., disintermediation—changing the way users get informed, debate, and shape their opinions [7–11] and confusion about causation may encourage speculation, rumors, and mistrust [12]. In 2011 a blogger claimed that Global Warming was a fraud aimed at diminishing liberty and democratic tradition [13], or even more recently, rumors about Ebola caused disruption to health-care workers [14–16]. Conspiracists tend to explain significant social or political aspects with plots conceived by powerful individuals or organizations having control of main stream media; their arguments can sometimes involve the rejection of science and invoke alternative explanations to replace scientific evidence. According to [12, 17], conspiracy theories are considered to belong to false beliefs overlooking the pervasive unintended consequences of political and social action.

Such a scenario presents an impressive amount of conspiracy-like narratives aimed at explaining reality and its phenomena, and provides an unprecedented opportunity to study the dynamics of narratives’ emergence, production, and popularity on social media.

Recently, we observed that the more users are exposed to unsubstantiated rumors, the more they are likely to jump the credulity barrier [5, 6, 18]. As pointed out by [19], individuals can be uninformed or misinformed, and the current means of corrections in the diffusion and formation of biased beliefs are not effective. In fact, corrections frequently fail to reduce misperceptions and, in several cases, they even strengthen them, acting as a *backfire effect* [20]. In particular, in [21] online debunking campaigns have been shown to create a reinforcement effect in usual consumers of conspiracy stories. Narratives grounded on conspiracy theories play a social role in simplifying causation because they tend to reduce the complexity of reality and are able at the same time to contain the uncertainty they generate [22–24]. In general, conspiracy thinking creates a climate of disengagement from mainstream society and from officially recommended practices [25]—e.g. vaccinations, diet, etc.

Despite the enthusiastic rhetoric about the *collective intelligence* [26, 27] the World Economic Forum listed massive digital misinformation as one of the main threats for our society [28]. According to this report the most discussed topics about misinformation relate to health, economy, climate change.

A multitude of mechanisms animates the flow and acceptance of false rumors, which, in turn, create false beliefs that are rarely corrected once adopted by an individual [29–32]. The factors behind the acceptance of a claim (whether documented or not) may be altered by normative social influence or by the coherence with the system of beliefs of the individual [33, 34] making the preferential driver of contents *confirmation bias*. A large body of literature addresses the study of social dynamics on socio-technical systems from social contagion up to social reinforcement [9, 10, 35–42].

Toward the understanding of the driving forces and dynamics behind the consumption and popularity of content as well as the emergence of narratives, in this work we analyze a collection of conspiracy news sources in the Italian Facebook over a time span of 4 years. We identify pages diffusing conspiracy news—i.e. pages promoting contents *neglected* by main stream media. We define the space of our investigation with the help of Facebook groups very active in debunking conspiracy theses (*Protesi di Protesi di Complotto*, *Che vuol dire reale*, *La menzogna diventa veritá e passa alla storia*). Conversely, science pages are active in diffusing posts about the most recent scientific advances. Pages are categorized according to their contents and their self description. We do not focus on the truth value of information but rather on the possibility to verify the content of the page. While the latter is an easy task for scientific news—e.g., by identifying the authors of the study or if the paper passed a peer review process—it usually becomes more difficult for conspiracy-like information, if not unfeasible. Through a semi-automatic topic extraction strategy, we find that the most discussed contents refer to four well specified semantic categories (or topics): environment, diet, health, and geopolitics. Contents belonging to the different categories (or topics) are consumed in a very similar way by their respective audience—i.e, users activity in terms of likes and comments on posts belonging to different categories are similar and resolves in comparable information consumption patterns. Conversely, if we focus on the lifetime –i.e., the distance in time between the first and the last comment for each user—we notice a remarkable difference within topics. Users polarized on geopolitics subjects are the most persistent in commenting, whereas the less persistent users are those focused on diet narratives. Finally, by analyzing mobility of users across topics, we find that users can jump independently from one topic to another, and such a probability increases with the user engagement. Users once inside the conspiracy corpus tend to join the overall corpus. This work provides important insights about the fruition of conspiracy like rumors in online social media and more generally about the mechanisms behind misinformation diffusion.

## Results and Discussion

The analysis aims at characterizing the topical space in the conspiracy corpus of the Italian Facebook. We start our investigation by outlining the emerging topics and then we focus on the information consumption patterns. Finally we provide a data-driven model of users information consumption patterns. Details about the mathematical and statistical tools as well as the data used in the analysis are described in Methods section.

### Topics extraction and validation

As a first step in our analysis we apply a semi-automatic topic extraction strategy aimed at classifying content. To avoid potential bias and misinterpretation of the language that is really specific of the community, we do not apply any lemmatization or stemming process to the conspiracy corpus.

We have 205, 703 posts (98.62% of the total corpus of conspiracy posts) containing a message—i.e. a simple text or a description of the associated photo, video, or link. We build a Document-Term matrix (205, 703 posts × 216, 696 terms) and take all the terms with more than 500 occurrences (1820). Then, we apply a supervised preprocessing in order to identify terms related to the conspiracy storytelling. Such a supervised task is performed by 20 volunteers separately. Notice that we consider as *conspiracy terms* only those terms labeled as conspiratorial by at least the 90% of volunteers. The resulting set is composed by 159 terms.

Then, we derive the co-occurrence network of conspiracy terms—i.e., a graph where nodes are conspiracy terms, edges bond two nodes if the corresponding terms are found in the same post, and weights associated to edges indicate the number of times the two terms appear together in the corpus. Such a graph has 159 nodes and 11, 840 edges.

**Fig 1:**
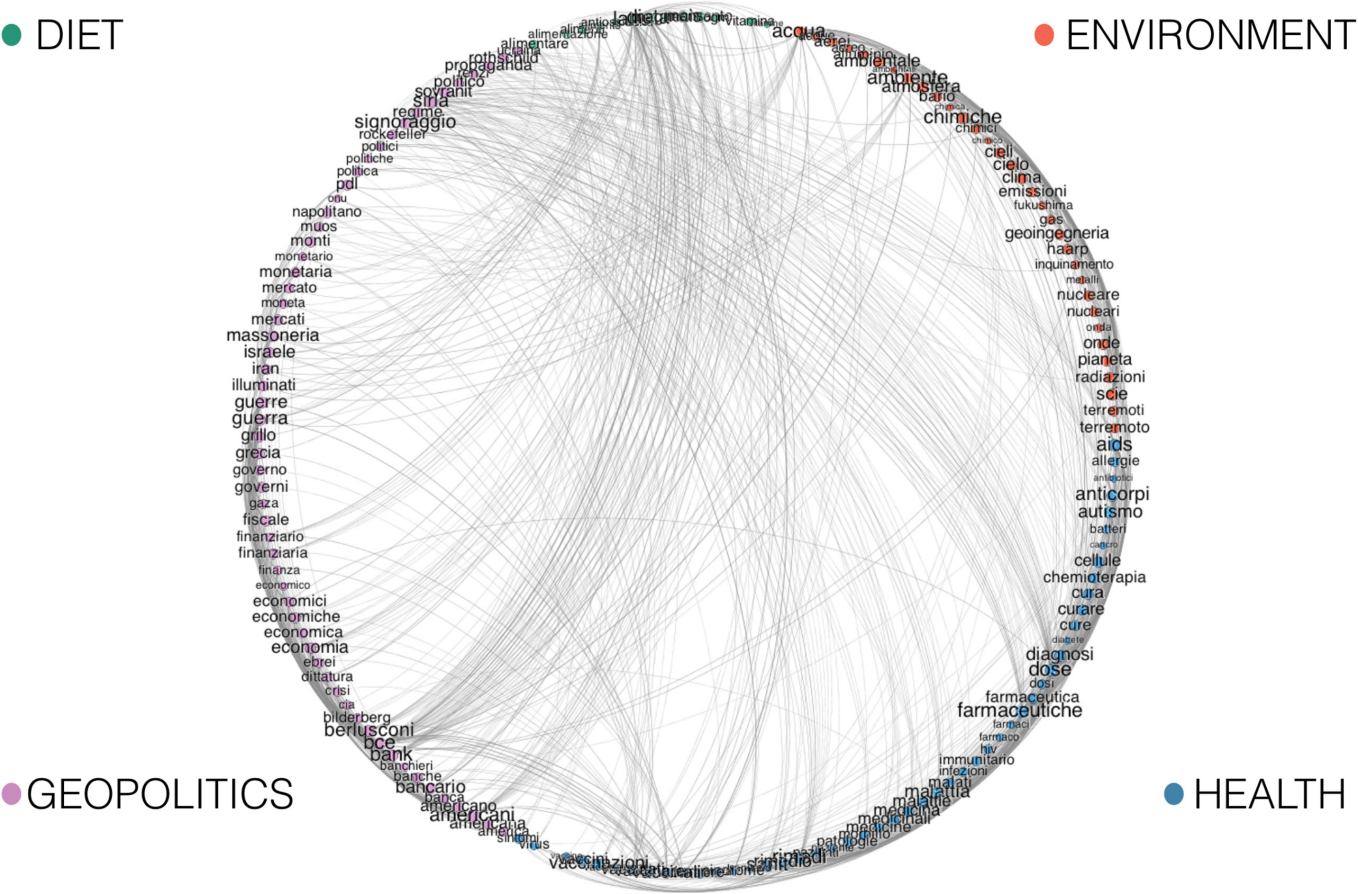
Backbone of conspiracy terms co-occurence network. Different colors indicate nodes belonging to different semantic category according to the output of the supervised tagging. In particular, purple nodes belong to geopolitics, red nodes to environment, blue nodes to health, and green to diet.

Since the co-occurrence network is a dense graph, we apply the disparity filter algorithm [43] (see Methods section for details) to extract the network backbone structure, thus reducing the number of edges while preserving its multi-scale nature. The application of the filtering algorithm with a statistical significance level of *α* = 0.05 results in a graph with 159 nodes and 1, 126 edges. We asked to the volunteers to provide a generic class to each term. By accounting only for 90% of concordance within volunteers semantic tags on terms, we identify four main semantic categories (or topics): *environment*, *health*, *diet*, and *geopolitics*. In Fig 1 we show the backbone of the co-occurrence term network, where different colors indicate nodes belonging to different conspiracy class.

To validate the classification, we apply three different community detection algorithms—i.e., Walktrap [44], Multilevel [45], and Fast greedy [46] (see Methods section for further details)—to the backbone of conspiracy terms co-occurence network.

**Fig 2:**
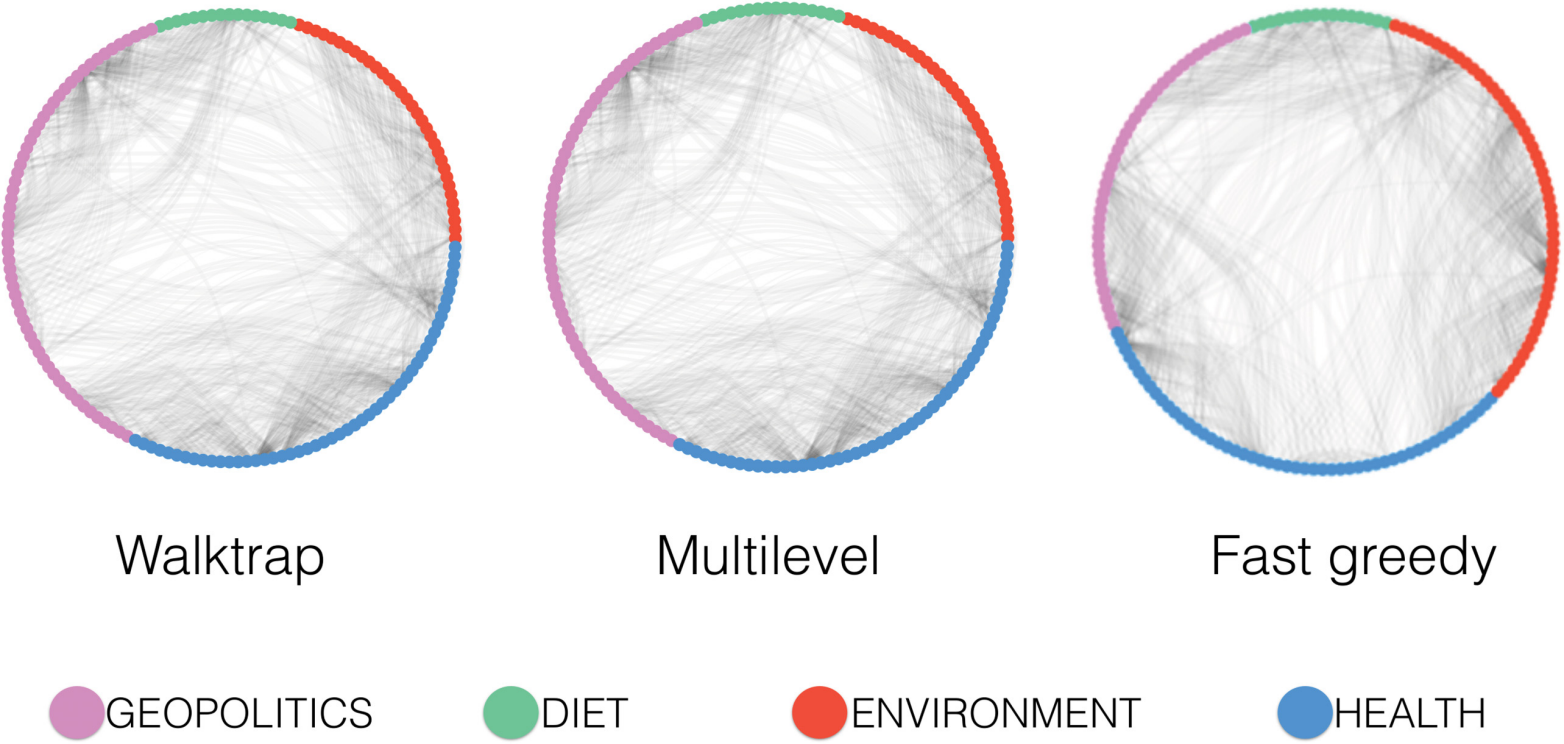
Communities of conspiracy terms. Membership of conspiracy terms according to different community detection algorithms. Purple nodes belong to geopolitics, red nodes to environment, blue nodes to health, and green to diet.

In Fig 2 we show the classification provided by each community detection algorithm. Multilevel and Walktrap algorithms assign each term to the same community and their accuracy with respect to our manual classification is 100%, while the concordance index of the Fast greedy algorithm is 88.68%.

We assign a post to a given topic according to the term in it. In case of terms belonging to different topics, we apply the majority rule, in case of ties, the post is not labeled. Through such a criterion we are able to label 44, 259 posts—i.e. 9, 137 environment posts, 8, 668 health posts, 3, 762 diet posts, and 22, 692 geopolitics posts.

### Attention patterns

#### Content consumption

In order to characterize how information belonging to the different categories are consumed, we perform a quantitative analysis on users’ interactions—i.e. likes, shares, and comments. Notice that each of these actions has a particular meaning [47]. A *like* stands for a positive feedback to the post; a *share* expresses the will to increase the visibility of a given information; and a *comment* is the way in which online collective debates take form around the topic promoted by posts. Comments may contain negative or positive feedbacks with respect to the post.

**Fig 3:**
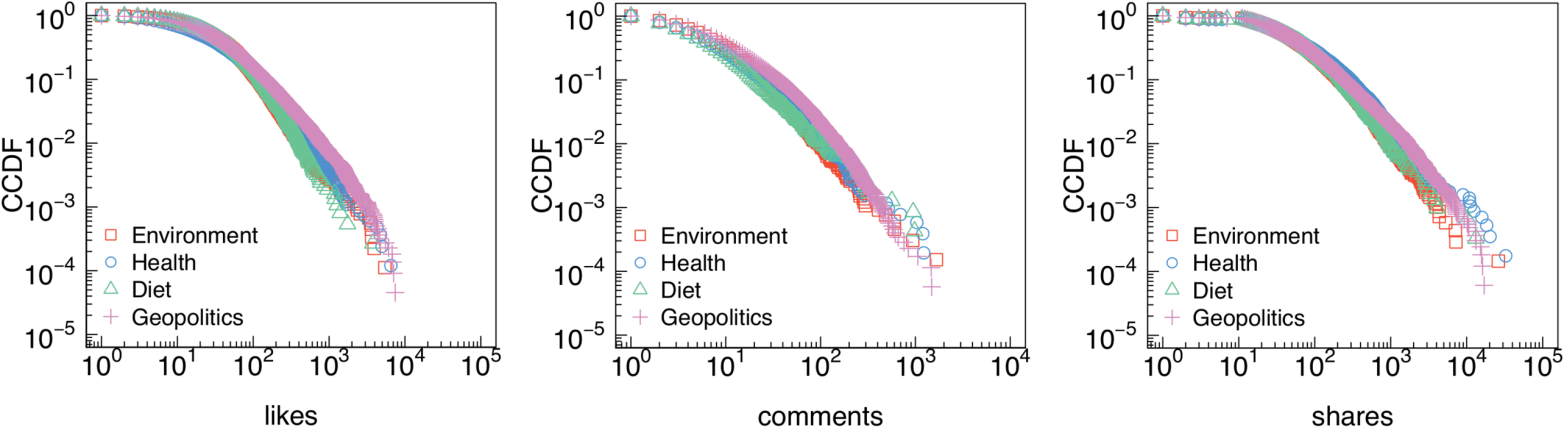
Attention patterns. Complementary cumulative distribution functions (CCDFs) of the number of likes (a), comments (b), and shares (c) received by posts belonging to different conspiracy semantic categories.

In Fig 3 we show the complementary cumulative distribution functions (CCDFs) of the number of likes, comments, and shares received by posts group by semantic category. All distributions are long-tailed and best fitted by a power law. Lower bounds and scaling parameters—i.e. how the tails of the distributions behave– have been estimated via minimization of Kolmogorov-Smirnov statistics and are shown in Table 1.

**Table 1:** Power law fit of conspiracy topics attention patterns. Lower bounds and scaling parameters estimates for the distributions of the number of likes, comments, and shares received by posts belonging to different conspiracy semantic category.

	**Likes**		**Comments**		**Shares**	
	${\hat{x}}_{min}$	$\hat{\alpha }$	${\hat{x}}_{min}$	$\hat{\alpha }$	${\hat{x}}_{min}$	$\hat{\alpha }$
**Environment**	142	2.82	42	2.82	408	2.62
**Health**	172	2.68	37	2.59	435	2.39
**Diet**	135	2.84	15	2.36	358	2.59
**Geopolitics**	167	2.36	135	3.14	407	2.25

**Fig 4:**
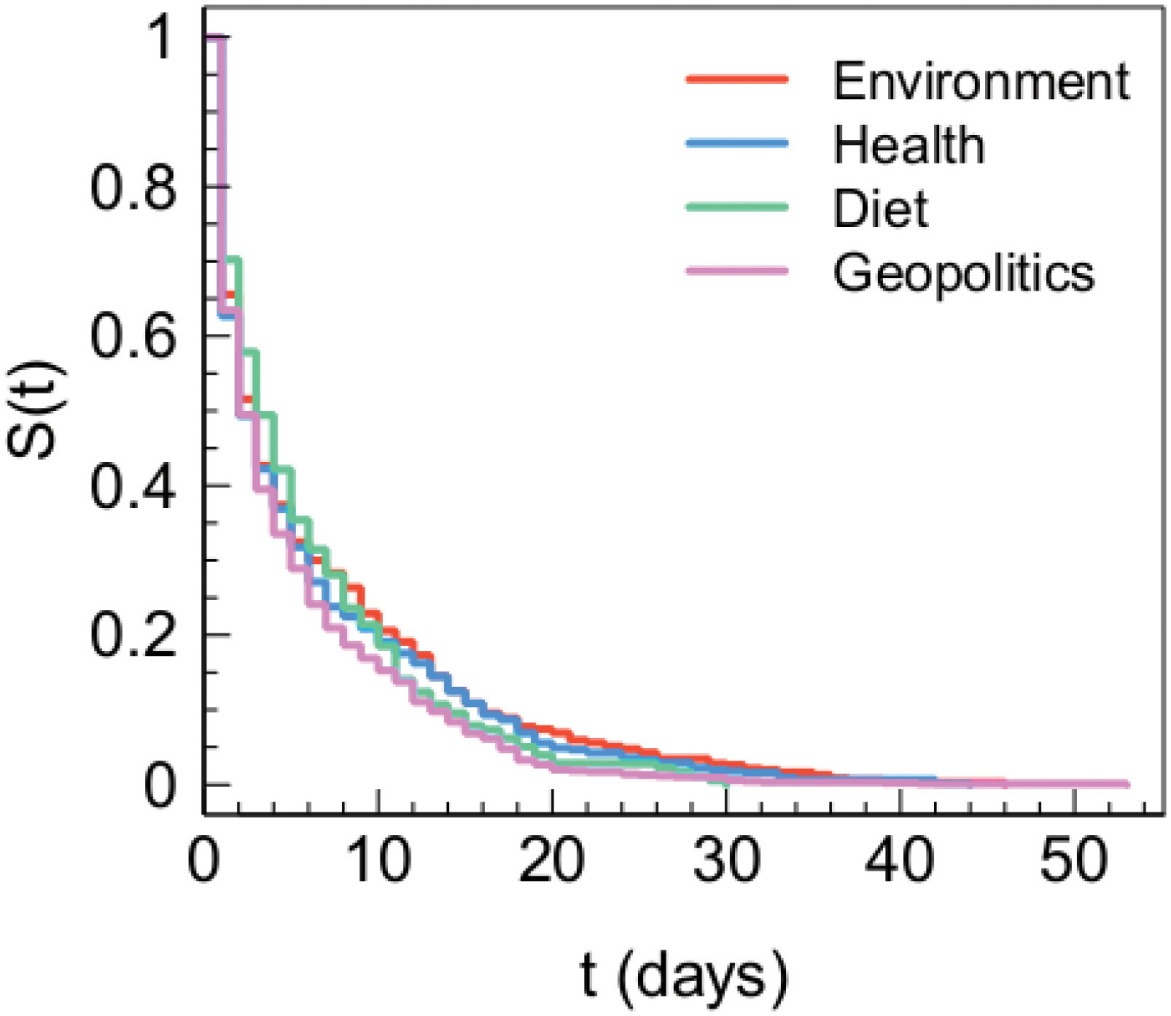
Lifetime of conspiracy topics. Kaplan-Meier estimates of survival functions of posts by posts belonging to different conspiracy semantic categories.

To analyze lifetime of posts from different categories, we compute the temporal distance between the first and last comment for each post. In Fig 4 we show the Kaplan-Meier estimates of survival functions (see Methods for further details) in the different semantic categories. The p-value associated to the Gehan-Wilcoxon test (a modification of the log-rank test) is *p* = 0.091, which lets us conclude that there are not significant statistical differences between the survival functions.

Our findings show that conspiracy matters are consumed in a similar way. In particular, we find that survival functions of posts belonging to different conspiracy topics do not show different statistical signatures.

#### Users activity

Here, we consider users’ attention patterns with respect to different conspiracy semantic categories by analyzing the number of likes and comments, as well as the lifetime of each user—i.e. the temporal distance between his first comment and last comment on a post belonging to a specific category—that can be intended as an approximation of users persistence in online collective debating.

We consider as conspiracy users those whose liking activity on conspiracy pages is greater than the 95% of their total liking activity—i.e., a conspiracy user left at most 5% of her likes on posts belonging to science pages. Such a method allows to identify 790, 899 conspiracy users. Moreover, we consider a user polarized towards a given conspiracy topic if she has more than the 95% of her likes on posts belonging to that topic. Such a criterion allows to classify 232, 505 users (29.39% of the total). Table 2 summarizes the classification task’s results. We observe that the majority of polarized users is concerned about conspiracy stories related to geopolitics (62.95%), whereas conspiracy narratives about environment (18.39%) and health (12.73%) attract a smaller yet substantial number of users, while diet (5.94%) seems to be considered a less attractive subject.

**Table 2:** Polarization of users towards different conspiracy semantic category.

	**Users**	**%**
**Geopolitics**	146, 359	62.95
**Environment**	42, 750	18.39
**Health**	29, 587	12.73
**Diet**	13, 807	5.94

**Fig 5:**
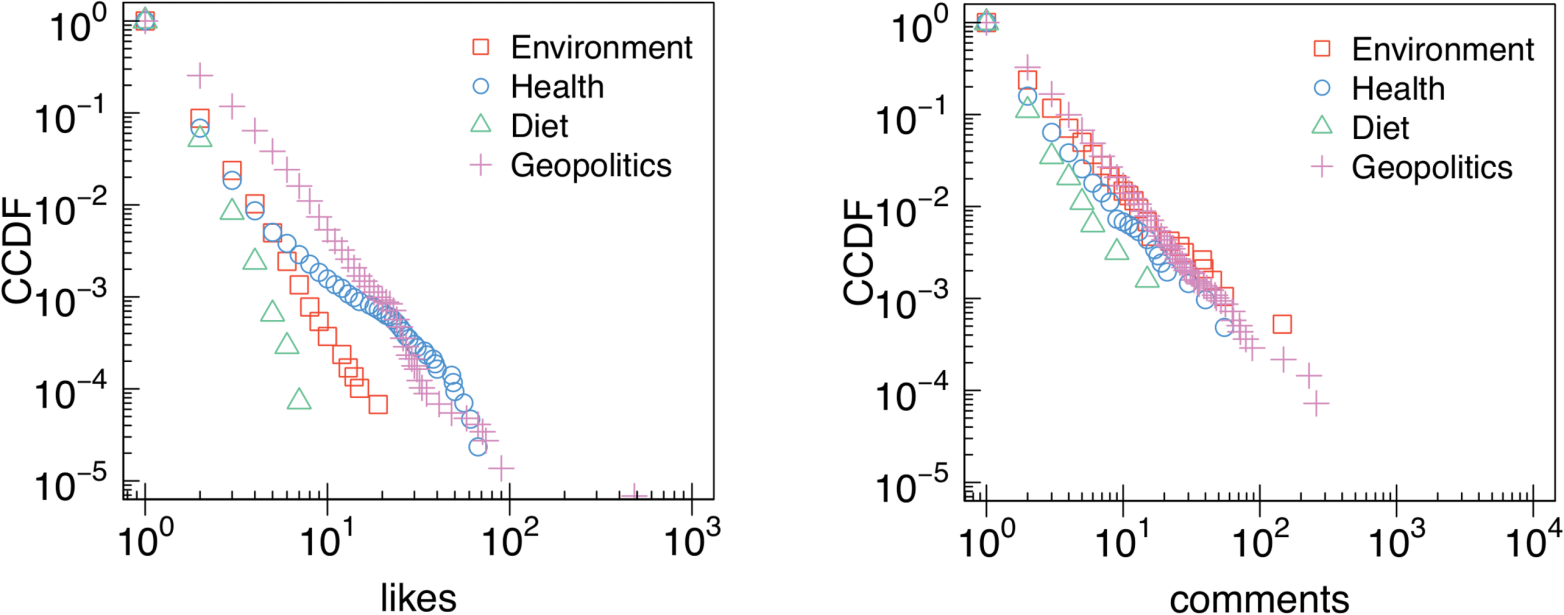
Users attention patterns. CCDFs of the number of likes (a) and comments (b) by users polarized on different conspiracy topics.

In Fig 5 we show the CCDFs of the number of likes and comments of users polarized towards different conspiracy category. We observe minor yet significant differences between attention patterns of different conspiracy users. Table 3 summarizes the estimated lower bounds and scaling parameters for each distribution. These results show that users polarized towards different conspiracy topics consume information in a comparable way—i.e, with some differences all are well described by a power law.

**Table 3:** Power law fit of conspiracy users attention patterns. Lower bounds and scaling parameters estimates for the distributions of the number of likes and comments left by users polarized towards different conspiracy semantic categories.

	**Likes**		**Comments**	
	${\hat{x}}_{min}$	$\hat{\alpha }$	${\hat{x}}_{min}$	$\hat{\alpha }$
**Environment**	5	4.37	3	2.49
**Health**	5	2.51	3	2.56
**Diet**	4	5.52	3	2.94
**Geopolitics**	6	3.61	6	2.88

**Fig 6:**
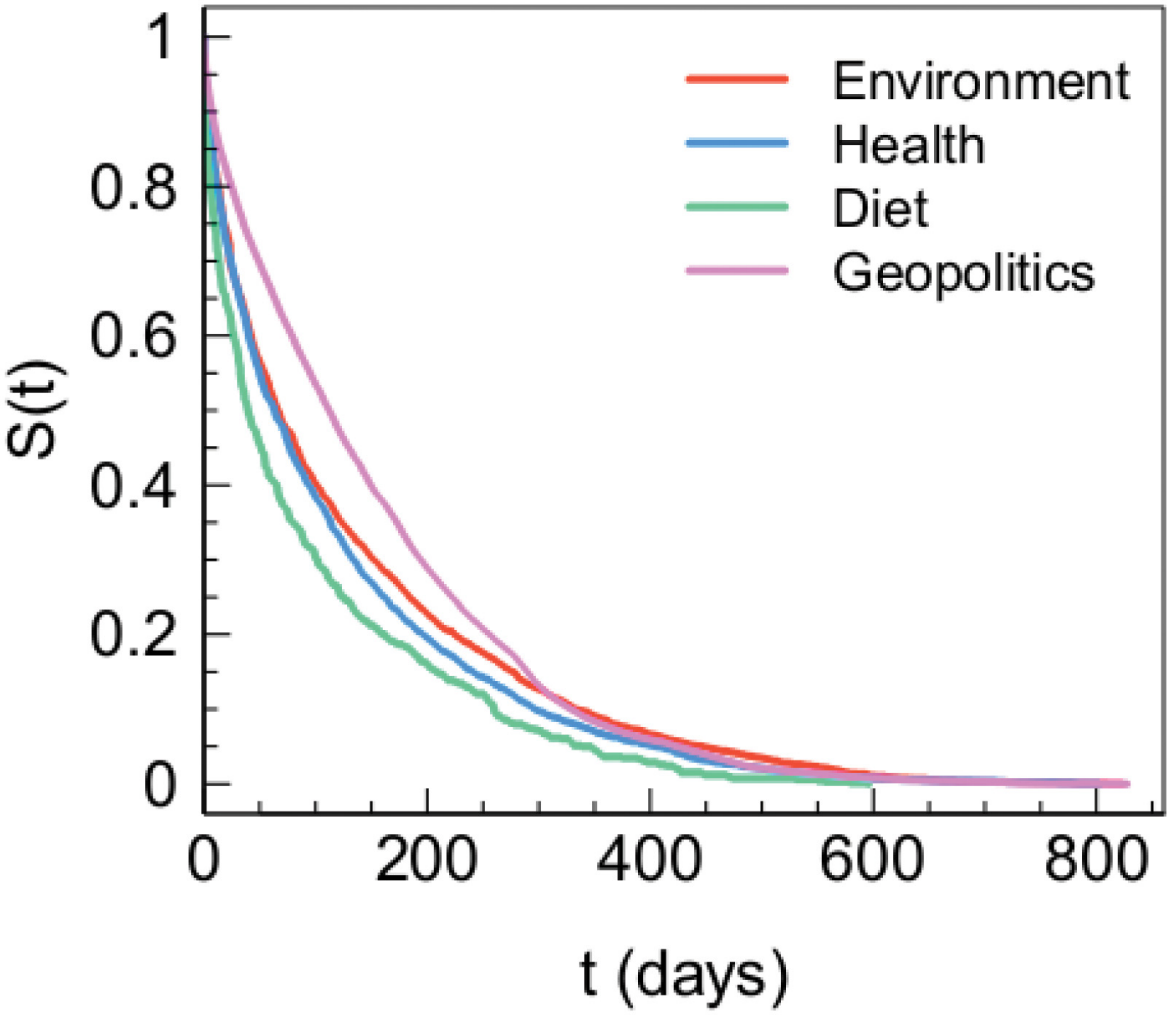
Persistence of conspiracy users. Kaplan-Meier estimates of survival functions for users polarized towards different conspiracy topics.

In order to analyze the persistence of polarized users, we compute the temporal distance between the first and last comment of each user on posts belonging to the specific category on which the user is polarized on. In Fig 6 we show the Kaplan-Meier estimates of survival functions (see Methods section for further details) for conspiracy users polarized towards different topics.

The Gehan-Wilcoxon test assesses a significant difference between the four survival functions (all p-values are less than 10^−6^).

Summarizing, we observe minor yet significant differences in the way users polarized persists in consuming their preferred contents. Moreover, by focusing on the lifetime—i.e. the temporal distance between users’ first and last comment—we find a remarkable difference within those users. In particular, we notice that users polarized on geopolitics subjects are the most persistent in commenting, whereas the less persistent users are those focused on diet related contents.

### Modeling user mobility

In this section we focus on users’ activity across different parts of the conspiracy corpus. In Table 4 we summarize users’ behavior by showing the Pearson correlations of their liking activity within the different categories of contents. We see meaningful correlations between the liking activity of users across the different semantic categories.

**Table 4:** Mobility of users across categories. Pearson correlations coefficients of conspiracy users’ liking activity between different categories.

	**Envir**	**Health**	**Diet**	**GeoPol**
**Envir**	1.00	0.68	0.61	0.64
**Health**		1.00	0.78	0.65
**Diet**			1.00	0.48
**GeoPol**				1.00

**Fig 7:**
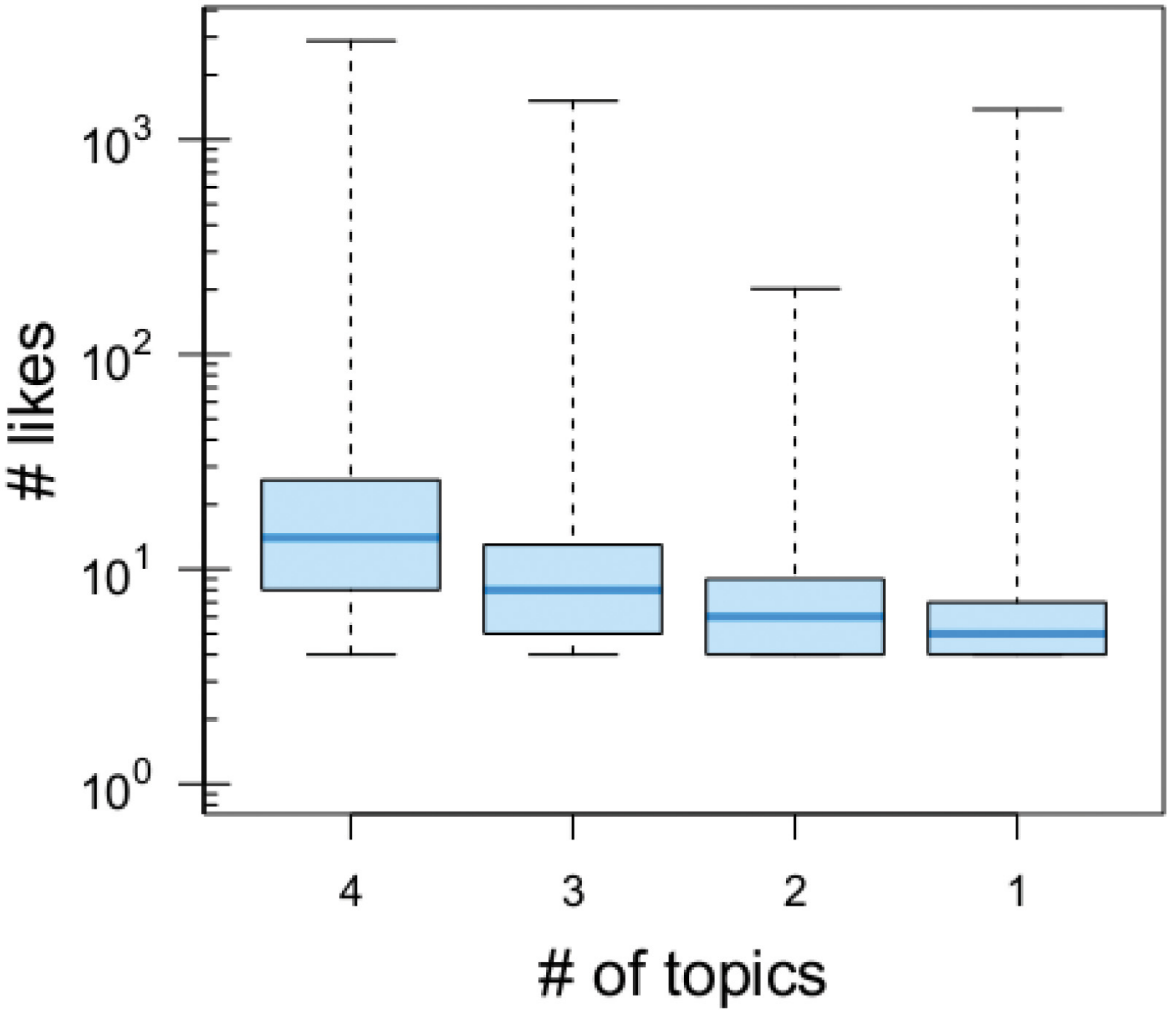
Engagement and mobility across semantic categories. Light blue lines represent the median of the likes distributions; pale blue shaded boxes represent the interquartile range (25–75 percentile); horizontal bars represent the extremes of the distributions. Users active on 4 categories are 15, 510; users active on 3 categories are 20, 929; users active on 2 categories are 21, 631; and users active on 1 category are 9, 980.

We analyze the relationship between the engagement of a user—i.e. the number of likes she left on conspiracy posts—and how her activity is distributed across categories. Fig 7 shows that the more a conspiracy user is engaged the more his activity spread on the overall corpus.

By considering only users with at least 4 likes (*n* = 68, 050)—necessary condition to be active on the four identified categories—we can model the relationship between the number of likes and the number of categories by means of a proportional odds model (see Methods section for an extended discussion).

In particular, we consider the number of categories liked by users as the ordinal dependent variable, i.e. we have *j* = (*K* − 1) = 3 ordered categories: 1∣2, 2∣3, and 3∣4. We consider the number of likes left by users as the predictor of our model. Thus, we need to estimate three intercepts and one regression coefficient. Table 5 reports details about the performed regression.

**Table 5:** Proportional Odds Model. Log-odds regression coefficient and intercepts with related standard errors, t-values, and p-values. Confidence interval at 95% for the estimated coefficient is (0.1121, 0.1161). Chi-Square test’s p-value is 1, so we do not reject the null hypothesis (*H*_0_: current model is good enough) and conclude that the model is a good fit.

**Coefficients**	**Value**	**Std. Error**	**t-value**	**p-value**
# of likes	0.1141	0.001016	112.3	< 10^−6^
**Intercepts**	**Value**	**Std. Error**	**t-value**	**p-value**
1∣2	-0.7602	0.0135	-56.4267	< 10^−6^
2∣3	1.0783	0.0126	85.7607	< 10^−6^
3∣4	2.9648	0.0177	167.4990	< 10^−6^

The estimated coefficient, *β*, can be difficult to interpret because it is scaled in terms of logs. Another way to interpret these kind of regression models is to convert the coefficient into a odds ratio (see Methods section for further details). To get the odds ratio (OR) we exponentiate the estimate:

$$\begin{array}{c} OR=\exp(\beta )=\exp(0.1141)=1.12 \end{array}$$

Since *OR* > 1, an increase in the number of likes left by a user raises her probability to consider a greater number of topics. In particular, an increase in the number of likes is associated with 12% times increased odds of considering a higher number of topics.

The model provides four probabilities for each user to belong to one of the four categories. For each user we consider the category associated with the higher probability as the predicted category. In order to evaluate the goodness of fit of the model, we compare the predicted categories vs the real ones by means of the absolute distance coefficient and we find that:

$$\begin{array}{c} \delta =1-\frac{\sum |d_{i}|}{n(K-1)}=0.852, \end{array}$$

where ∣*d*_*i*_∣ = ∣*x*_*i*_ − *y*_*i*_∣ is the absolute distance between the real and the predicted categories, *n* is the total number of users, and *K* is the number of categories. Since the absolute distance coefficient is close to 1, the proposed model provides a good fit for the data.

Summarizing, the more a user is engaged in conspiracy storytelling the more her probability to consider a higher number of different conspiracy topics. Indeed, we deliver a data-driven model of information consumption pointing out that users engagement on different topics is mainly driven by their overall commitment on conspiracy storytelling and that with the increasing of the engagement they tend to span on the overall corpus.

## Conclusions

Conspiracy theories are considered to belong to false beliefs overlooking the pervasive unintended consequences of political and social action. Social media fostered the production of an impressive amount of rumors, mistrust, and conspiracy-like narratives aimed at explaining (and oversimplifying) reality and its phenomena. Such a scenario provides an unprecedented opportunity to study the dynamics of topics emergence, production, and popularity. Indeed, in this work we focus on how conspiracy contents are consumed in the Italian Facebook.

The understanding of consumption patterns behind unsubstantiated claims might provide important insight both at the level of popularity of topics as well as to prevent misinformation spreading. Users activity in terms of likes and comments on posts belonging to different categories are similar and resolves in similar information consumption patterns. Conversely, if we focus on the lifetime—i.e., the distance in time between the first and the last comment for each user—we notice a remarkable difference within topics. Users polarized on geopolitics subjects are the most persistent in commenting, whereas the less persistent users are those focused on diet narratives. Finally we focus on the mobility of users across the different semantic categories. In particular, we address the patterns behind the consumption of different topics with respect to the user’s engagement. Previous works [4, 5] showed that users tend to aggregate around their preferred information and build their own narrative in social echo-chambers. In particular, conspiracy users resulted to be more focused and self-contained on their specific contents. Here we find that, in their own echo-chamber, users can jump independently from one semantic category to another, and such a probability increases with the user engagement (number of likes on a single specific category). Each new like on the same category increases of the 12% the probability to pass to a new one.

## Methods

### Ethics Statement

The entire data collection process has been carried out exclusively through the Facebook Graph API [48], which is publicly available, and for the analysis (according to the specification settings of the API) we used only public available data (users with privacy restrictions are not included in the dataset). The pages from which we download data are public Facebook entities (can be accessed by anyone). User content contributing to such pages is also public unless the user’s privacy settings specify otherwise and in that case it is not available to us.

### Data Collection

We define the space of our investigation with the help of some Facebook groups very active in the debunking of conspiracy theses. The resulting dataset is composed by 39 public Italian Facebook pages.

Notice that the dataset is the same used in [5] and [21]. However, in this paper we focus on 39 (exhaustive set) conspiracy pages aiming at characterizing attention dynamics driving the diffusion of conspiracy topics on the Italian Facebook. We download all posts from these pages in a timespan of 4 years (2010 to 2014). In addition, we collect all the likes and comments from the posts, and we count the number of shares. In Table 6 we summarize the details of the data collection.

**Table 6:** Breakdown of the Facebook dataset.

**Entity**	**Total**
Pages	39
Posts	208, 591
Likes	6, 659, 382
Comments	836, 591
Shares	16, 326, 731
Likers	864, 047
Commenters	226, 534

### Preliminaries and Definitions

#### Bipartite Network

We consider a bipartite network whose nodes are conspiracy posts and conspiracy terms. The presence of a term on a given post determines a link between the term and the post. More formally, a bipartite graph is a triple 𝒢 = (A, B, E) where *A* = {*a*_*i*_ ∣ *i* = 1, …, *n*_*A*_} and *B* = {*b*_*j*_ ∣ *j* = 1, …, *n*_*B*_} are two disjoint sets of nodes, and *E* ⊆ *A* × *B* is the set of edges—i.e. edges exist only between nodes of the two different sets *A* and *B*. The bipartite graph 𝒢 is described by the matrix *M* defined as

$$M_{ij}=\{\begin{array}{cc} 1 & if\;an\;edge\;exists\;between\;a_{i}\;and\;b_{j}\\ 0 & otherwise \end{array}$$

Referring to *A* as the set of conspiracy terms, in our analysis we use the co-occurrence matrix *C* ^*A*^ = *MM* ^*T*^, that is the weighted adjacency matrix of the co-occurrence of conspiracy terms on conspiracy posts. Each non-zero element of *C* ^*A*^ corresponds to an edge among nodes *a*_*i*_ and *a*_*j*_ with weight $P_{ij}^{A}$.

#### Disparity Filter

Disparity filter is a network reduction algorithm which extracts the backbone structure of a weighted network, thus reducing its size without destroying its multi-scale nature. In particular, the method introduced in [43] is based on the null hypothesis that the normalized weights corresponding to the connections of a given node with degree *k* are uniformly distributed. The disparity filter identifies which links for each node should be preserved in the network. The null model allows such a discrimination through the computation—for each edge of a given node—of the probability *α*_*ij*_ that its normalized weight *p*_*ij*_ is compatible with the null hypothesis. All the links with *α*_*ij*_ smaller than a certain significance level *α* reject the null hypothesis, and can be considered as significant heterogeneities characterizing the network. The statistically significant edges will be those whose weights satisfy the relation

$$\begin{array}{c} \alpha_{ij}=1-\left(k-1\right)\int_{0}^{p_{ij}}{\left(1-x\right)}^{k-2}dx<\alpha , \end{array}$$

indicating that by decreasing the significance level *α* we can filter out additional links, and thus focus on more relevant edges.

#### Community Detection Algorithms

In order to validate our manual classification of conspiracy terms, we apply three well known community detection algorithms to the backbone of the conspiracy terms co-occurrence network.

Walktrap [44] computes a measure of similarities between nodes based on random walks which has several important advantages: it captures well the community structure in a network, it can be computed efficiently, and it can be used in an agglomerative algorithm to compute efficiently the community structure of a network. Such an algorithm runs in time $𝒪\text{(}mn^{\text{2}}\text{)}$ and space $𝒪\text{(}n^{\text{2}}\text{)}$ in the worst case, and in time $𝒪\text{(}n^{\text{2}}\text{ log }n\text{)}$ and space $𝒪\text{(}n^{\text{2}}\text{)}$ in most real-world cases, where *n* and *m* are respectively the number of nodes and edges in the network.

Multilevel [45] is based on multilevel modularity optimization. Initially, each node is assigned to a community on its own. In every step, nodes are re-assigned to communities in a local, greedy way. Nodes are moved to the community in which they achieve the highest modularity. Such an algorithm runs in linear time when *m* ∼ *n*, where *n* and *m* are respectively the number of nodes and edges in the network.

Fast greedy [46] it is a hierarchical agglomeration algorithm for detecting community structure. Its running time on a network with *n* nodes and *m* edges is 𝒪(md log n) where *d* is the depth of the dendrogram describing the community structure. Many real-world networks are sparse and hierarchical, with *m* ∼ *n* and *d* ∼ log *n*, in which case such an algorithm runs in essentially linear time, $𝒪\text{(}n\;{\text{log}}^{\text{2}}\;n\text{)}$.

#### Kaplan-Meier estimator

Let us define a random variable *T* on the interval [0, ∞), indicating the time an event takes place. The cumulative distribution function (CDF), *F*(*t*) = **Pr**(*T* ≤ *t*), indicates the probability that such an event takes place within a given time *t*. The survival function, defined as the complementary CDF. We remind that the CCDF of a random variable *X* is one minus the CDF, the function *f*(*x*) = **Pr**(*X* > *x*).) of *T*, represents the probability that an event lasts beyond a given time period *t*. To estimate this probability we use the *Kaplan–Meier estimator* [49].

Let *n*_*t*_ denote the number of that commented before a given time step *t*, and let *d*_*t*_ denote the number of users that stop commenting precisely at *t*. Then, the estimated survival probability after time *t* is defined as (*n*_*t*_ − *d*_*t*_)/*n*_*t*_. Thus, if we have *N* observations at times *t*_1_ ≤ *t*_2_ ≤ ⋯ ≤ *t*_*N*_, assuming that the events at times *t*_*i*_ are jointly independent, the Kaplan-Meier estimate of the survival function at time *t* is defined as

$$\begin{array}{c} \hat{S}\left(t\right)=\prod_{t_{i}<t}\left(\frac{n_{t_{i}}-d_{t_{i}}}{n_{t_{i}}}\right). \end{array}$$

#### Odds ratio

Probability and odds are two basic statistical terms to describe the likeliness that an event will occur. Probability is defined as the fraction of desired outcomes in the context of every possible outcome with a value in [0, 1], where 0 would be an impossible event and 1 would represent an inevitable event. Conversely, odds can assume any value in [0, ∞), and they represent a ratio of desired outcomes versus undesired outcomes. Given a desired outcome *A*, the relationship between the probability *P*(*A*) that event *A* will occur, and its odds *O*(*A*) is

$$\begin{array}{c} P\left(A\right)=\frac{O(A)}{1+O(A)}\;and\;O\left(A\right)=\frac{P(A)}{1-P(A)}. \end{array}$$

It follows that the odds ratio (OR) of two events *A* and *B* is defined as

$$\begin{array}{c} OR\left(A,B\right)=\frac{O(A)}{O(B)}=\frac{\frac{P(A)}{1-P(A)}}{\frac{P(B)}{1-P(B)}}=\frac{P(A)[1-P(B)]}{P(B)[1-P(A)]}. \end{array}$$

#### Proportional Odds Model

The proportional odds model is a class of generalized linear models used for modeling the dependence of an ordinal response on discrete or continuous covariates.

Formally, let *Y* denote the response category in the range 1, …, *K* with *K* ≥ 2, and let *π*_*j*_ = **Pr**(*Y* ≤ *j* ∣ *x*) be the cumulative response probability when the covariate assumes value *x*. The most general form of linear logistic model for the *j*th cumulative response probability,

$$\begin{array}{c} logit\left(\pi_{j}\right)=ln(\frac{\pi_{j}}{1-\pi_{j}})=\alpha_{j}+\beta_{j}^{T}x, \end{array}$$

is one in which both the intercept *α* and the regression coefficient *β* depend on the category *j*. The proportional odds model is a linear logistic model in which the intercepts depend on *j*, but the slopes are all equal, i.e.

$$\begin{array}{c} logit\left(\pi_{j}\right)=ln(\frac{\pi_{j}}{1-\pi_{j}})=\alpha_{j}+\beta^{T}x. \end{array}$$

In other words, proportional odds model takes logistic regression one step further in order to account for ordered categorical responses. For instance, in our analysis we could have used a logistic regression model to investigate the effect of the number of comments on the odds ratio (OR) of “considering < 3 topics” vs “considering ≥ 3 topics”. However, in such a case the cut-point would be arbitrary, and we could have used a similar logistic regression model to analyze the effect on the odds ratio (OR) of “considering < 2 topics” vs “considering ≥ 2 topics”. In this sense, proportional odds model averages up over all possible cut-point logistic regression models to maximize the amount of information one can get out of the data.
